# Pregnancy risks and contraceptive use among postpartum mothers in Cameroon: implications for improving the coverage of postpartum family planning services

**DOI:** 10.1186/s12978-022-01552-1

**Published:** 2023-01-02

**Authors:** Jean Christophe Fotso, John G. Cleland, Marquise Kouo Ngamby, Martina Lukong Baye, Elihouh O. Adje

**Affiliations:** 1 EVIHDAF, Nouvelle Route Bastos, BP 35328 Yaoundé, Cameroon; 2 Department of Population Health, London School of Hygiene & Tropical Medicine, London, UK; 3 UNFPA Cameroon, Yaoundé, Cameroon; 4 National Multisector Program to Combat Maternal, Newborn & Child Mortality, Ministry of Health, Yaoundé, Cameroon; 5 EVIHDAF, Parakou, Benin

**Keywords:** Postpartum family planning, Pregnancy risks, Cameroon

## Abstract

**Background:**

The health hazards of short inter-birth intervals are severe in Cameroon. One-quarter of inter-birth intervals are less than 24 months and the probability of death before age 5 for children born after a short interval is double that associated with intervals of 36–47 months. We examine the risk of an unintended pregnancy in the 18 months following childbirth in Cameroon, taking into account the protective effects of lactational amenorrhea, delayed resumption of sex as well as contraceptive use.

**Methods:**

Data from 3007 postpartum women in the nationally representative 2018 Cameroon Demographic and Health Survey were used. Risk of an unintended pregnancy was defined from current status information on resumption of sex and menses, contraceptive use, desire for another child within 12 months, and, for the minority of pregnant women, whether the conception was intended. Predictors of risk, and of modern method use, were assessed by bivariate and multivariate analysis.

**Results:**

In the first 6 postpartum months, only 8% of women were fully at risk (i.e., sex and menses resumed but no contraceptive use), rising to 24% at 6–11 postpartum months, and further to 30% at months 12–17. Though 89% wanted to delay the next birth by at least 1 year, only 17% were currently using a modern method. Menstruating women were much more likely to be users than amenorrheic women: 27% versus 15% at months 12–17 postpartum. Urban and better educated women recorded higher contraceptive use but lower protection from other factors than rural, less educated women, with the net result that risk differed little across these population strata. Uptake of maternal and child health (MCH) services was high but only one-third of women had discussed family planning at a facility visit during the preceding 12 months.

**Conclusions:**

These results underscore the need for improved postpartum family planning services by means of closer integration with mainstream health services. In view of evidence from other sources of heavy workload and weak motivation of health staff, this will require strong leadership. A related priority is to increase the number of staff trained in provision of long-acting methods, such as implants.

## Plain Language Summary

We use information from mothers with a child under the age of 18 months, who were interviewed in a national survey conducted in 2018. We examine risk of an unintended pregnancy, using data on factors that protect against risk, namely delayed resumption of sex and menses, breastfeeding and contraceptive use. Among mothers with an infant aged less than 6 months, very few were at risk mainly because they had not resumed sex. Among those with an infant aged 6–11 months, 26% had still not resumed sex and an equal proportion was partially protected by delayed resumption of menses. Only 17% were protected by use of a modern contraceptive method, leaving 24% fully at risk. Among those with a child aged 12–18 months, 30% were fully at risk. As expected, well educated, urban women were more likely to use contraception than less privileged women but less likely to be protected by delayed resumption of sex and menses, with the consequence that pregnancy-risk was similar. Though three-quarters of mothers had taken their child for vaccination on three or more occasions, only one-third had discussed family planning with a health provider at a visit to a facility in the previous 12 months. The need for improved contraceptive services for mothers with young children is clear. Short intervals between births are common in Cameroon and these threaten the health of mothers and children. It is equally clear that closer integration of family planning into mainstream health services is needed.

## Background

The past decade has seen a resurgence of interest in postpartum family planning, partly in response to recommendations for programming strategies published by the World Health Organization (WHO) [1]. The rationale for this focus is threefold. First, the risk to the health and survival of closely spaced children is well established. A recent analysis, based on 4.5 million births from 77 countries, controlling for unobserved heterogeneity by using within-family models, supported the WHO recommendation that births should be spaced almost 3 years apart. Moreover, adverse effects of short inter-birth intervals were found to be most severe in high fertility counties at low levels of development [2]. Short intervals also pose risks for the mother including obstructed labor, hemorrhage, and hypertensive disorders, though no clear association with maternal death has been established [3]. Second, the subjective desire for pregnancy prevention is high following a birth, as a large majority of couples wish to delay the next pregnancy for 2 years or more [4]. Third, contact between women and health services is most intense during pregnancy, delivery, and in the 12 months following birth. Antenatal, delivery, and infant care services all offer opportunities for family planning counselling and contraceptive methods can be provided immediately after delivery or later during the postpartum period, for instance when mothers bring infants for immunization. All contraceptive methods can be initiated immediately following birth or after a delay of 6 weeks, with the exception of combined oral contraceptives for breast feeding women.

Postpartum contraceptive services need to consider the fact that lactational amenorrhea also offers protection against pregnancy. This protection was recognised by WHO and other family planning agencies in 1998 in the form of the Lactational Amenorrhea Method (LAM). LAM is acknowledged as an effective method under the following conditions: the mother is fully breastfeeding with little or no supplementary feeding, is amenorrheic, and her infant is less than 6 months old [1]. It can be argued that these conditions are too restrictive. A review of nine studies concluded that the risk of pregnancy in the 12 months after birth is, on average, 6% for sexually active, breastfeeding, amenorrheic women [5]. This risk is about the same as the failure rate for oral contraception and lower than the rate for condoms. There is, however, an important distinction. Pregnancy while using oral contraceptives or condoms is largely attributable to incorrect or inconsistent use, while pregnancy during lactational amenorrhea reflects the fact that first ovulation typically precedes resumption of menses, and thus cannot be anticipated or the risk of conception reduced by behavioural precautions. Though one study found that socio-economic development weakens the contraceptive effect of breastfeeding, presumably through the pathway of improved maternal nutrition, the risks of pregnancy during lactational amenorrhea in the first six postpartum months were found to be less than 2% in a study of well-nourished women despite supplementation [6, 7].

While it is uncertain whether women are aware of the risks of relying on lactational amenorrhea, it is firmly established that many in low- and middle-income countries delay contraceptive uptake until the return of menses. An analysis of survey data from 17 countries, mostly in sub-Saharan Africa, found that contraceptive use was typically two to three times more prevalent at six to nine months after birth among women whose menses had returned than among those still amenorrheic [8].

Though the standard Demographic and Health Survey (DHS) measure of unmet need considers delayed resumption of menses, most analyses of postpartum contraception follow the restrictive WHO approach which advocates contraceptive adoption except for those observing LAM, and derive high estimates of unmet need in the 12 months following birth [4, 9]. A systematic review and meta-analysis of postpartum contraception found that use of a modern contraceptive method was lowest in West Africa, at 36%, and unmet need highest at 59% [10]. Another systematic review investigating factors that influence postpartum contraceptive use and unmet need in sub-Saharan Africa reported that use of maternity services and receipt of family planning counselling were associated with reduced unmet need. Major reasons for non-use of contraception included fear of side effects, husband’s disapproval, absence of menses, delayed resumption of sex, and low perceived risk of pregnancy [11].

None of the papers cited in the previous paragraph included Cameroon. Indeed, little is known about postpartum contraception in this country. We located only one relevant study, which examined knowledge and attitudes based on a small sample of 300 respondents in one district [12]. Accordingly, the broad aim of this paper is to remedy this lack of evidence and provide guidance on ways in which postpartum family planning services can be improved. The specific objectives are to: (1) establish the level of exposure to the risk of an unintended pregnancy in the 18 months following childbirth, taking into account the protective contribution of sexual abstinence and amenorrhea as well as use of contraception; (2) identify population groups most at risk; (3) investigate the factors associated with use of a modern method of contraception in the extended postpartum period; and finally (4) explore opportunities for increasing postpartum contraceptive use by documenting the extent to which mothers have received recent counselling on family planning and whether receipt is associated with contraceptive use.

### Context

Cameroon has an ethnically diverse population of about 26 million. Between 1990 and 2019 life expectancy rose from 53 to 59 years, mean years of schooling from 3.5 to 6.3, gross national income per head (at 2017 PPP$) from 3,100 to 3580, and the human development index from 0.448 to 0.563 [13]. According to the Cameroon Demographic and Health Surveys (CDHSs), the maternal mortality ratio dropped by 40% between 2011 and 2018, from 782 to 406 deaths per 100,000 livebirths. The 2018 CDHS found that 67% of recent births took place in a health facility; infant mortality fell from 70 deaths per thousand births in the 1990s to 48 in the recent past. The percent of children receiving the full schedule of vaccinations rose from 40 to 52% over the same period. CDHSs also document a fall in HIV prevalence from 6.6% in 2004 to 3.4% in 2018 among in women aged 15 to 49, and from 4.1% to 1.9% among men in the same age bracket. Likewise, a more recent study revealed that HIV incidence has fallen dramatically since its peak of 500 cases per 100,000 population in the late 1990s [14].

CDHS reports show that despite these positive trends, the total fertility rate (TFR) has declined at a slow pace from slightly over six births per woman in the mid-1980s to just below five births per woman in 2015–18, with a large rural–urban contrast of about 6.0 in rural areas compared with 3.8 in urban localities. The level of modern contraceptive use in married women remains low at 15% in 2018, with little change since 2004. Total demand for family planning in married women actually fell from 46 to 42% between 2012 and 2018. However, modern method use is much higher, at 43% in 2018, among sexually active unmarried women. The main driver of fertility decline has probably been delayed marriage and first birth, together with increased uptake of contraception before marriage, rather than falls in marital fertility.

Birth intervals and postpartum behaviour are of particular relevance to the purposes of this paper. Unlike many other African countries, the length of birth intervals in Cameroon has remained unchanged since 1991, the date of the first CDHS, with a median length of about 30 months. Similarly, the percentage of inter-birth intervals that are less than 24 months has fluctuated over time between 21 and 25%. The combination of high fertility and short birth intervals underscores the important potential contribution of postpartum contraception to further improvement in child health and survival. In a country with a TFR of two births, only half of children are exposed to the risk of a short preceding birth interval, whereas in Cameroon, with a TFR of close to 5, four-fifths (i.e., 80%) of children have an elder sibling and are thus potentially exposed.

The risk to child survival of short intervals is severe in Cameroon. The 2018 CDHS reveals a sharp gradient in under-five mortality. For children born within 24 months of an elder sibling, 135 per thousand died before their fifth birthday. This figure falls to 92, 67 and 47 for children born after intervals of two, three and four or more years, respectively.

While births reported by mothers in Cameroon as unwanted are rare, at 4% in the 2018 CDHS, 19% were reported as mistimed and this proportion has changed only minimally since 1991. This estimate constitutes strong evidence of a demand for improved birth spacing and thus a need for improved postpartum contraception.

Postpartum behaviours—breastfeeding and delayed resumption of sex—are major determinants of birth interval length. In Cameroon, prolonged breastfeeding remains the norm; the median length of lactation declined only slightly between 1991 and 2018, from 17 to 15 months. The relative decline in postpartum amenorrhea was more pronounced, from 10.5 to 8.3 months. The most emphatic change concerns postnatal sexual abstinence, which fell from 13 months in 1991 to 4.2 months in 2018. This trend is likely to be associated with the decline in polygyny, with the proportion of married women with co-wives falling from 39 to 25% over the same period.

### Family planning services

Over the past 40 years, Cameroon government policy on population has shifted gradually from a pronatalist to a cautious anti-natalist stance. Population policies, initially adopted in 1993 and revised in 2002, were characterised by broad-based goals with little reference to demographic topics and no quantifiable targets [15].

Policies on family planning have strengthened in the past decade. In 2012, as part of the fulfilment of its FP2020 commitments, Cameroon adopted a budget line for the purchase of contraceptives in order to improve the availability of contraceptives, but these funds could not be mobilized, due to competing priorities for government funding [16]. The country developed its 2015–2020 operational plan which describes high-impact interventions to increase contraceptive prevalence from 16.1% to 30%. The Plan targeted about 1.8 million new users at the national level [17]. No doubt in response to these developments, Cameroon’s National Composite Index for Family Planning, which measures the strength of family planning policy and implementation, rose from a score of 51 in 2014 to 67 in 2018 [18]. However, there is little sign, so far, that contraceptive use has increased. According to CDHSs, the prevalence of use of a modern method by married women remained unchanged at 15–16% between 2011 and 2018 and reported use among sexually active unmarried women actually fell. Though use of injectables and implants has gradually increased, condoms, typically obtained from commercial sources, remain the most commonly used modern method, even among married women.

In Cameroon, the Family Health Department within the Ministry of Health is responsible for family planning. Services are offered by health personnel trained in contraceptive technology, either by pre-service or continuous training. According to the level of the health facility, family planning is offered either by a dedicated unit or within the framework of integrated services. In general, staff who offer family planning in health facilities are insufficient in number. Though accurate data are not available on the number of personnel trained in intrauterine device (IUD) and Implant insertions, a severe shortage of such staff has been acknowledged by the government and its partners as a major barrier to family planning service delivery in the country.

Limited information on postpartum family planning comes from a project and quasi-experiment, implemented by Evidence to Action (E2A), led by Management Sciences for Health (MSH) with funding from the United States Agency for International Development (USAID). The quasi-experiment involved six hospitals in Yaoundé and the effect on contraceptive uptake was measured in terms of couple-years of protection (CYPs) over a three-month period prior to, and following, the intervention. The effect was positive but very modest. In hospitals receiving management and clinical training, CYPs rose from 23.8 to 33.1 and from 20.8 to 35.0 in hospitals receiving clinical training only [19]. Perhaps for this reason, the project failed in its intention to become a basis for widespread scale-up. Many barriers to postpartum family planning were identified, including lack of demand, supply-chain defects, weak staff motivation, heavy obstetric work load, and inability of some patients to pay fees. More recent experimental evidence indicates that offering discounts on the full fee for IUDs and implants (CFA 4000 = US$ 7.25) can increase uptake, including among postpartum women [20].

Both long-acting reversible contraceptives (LARCs) and short-acting methods are part of essential drugs and their availability is the responsibility of the national centre for supply of essential drugs and medical consumables. Moreover, the procurement of contraceptives is done at the global level with the support of the United Nations Population Fund (UNFPA) and other partners, on the basis of national quantification. At the level of health facility, despite the subsidization of cost in order to improve financial access to long-acting methods, their availability remains low due to a poorly functional logistics chain and insufficient demand creation. Accessibility to family planning services, particularly injectables and including Sayana Press (DMPA-SC), has been improved by community-based distribution through trained community health workers.

In a bid to expand services, social franchising and social marketing strategies are also implemented in Cameroon. Population Services International (PSI)’s Cameroon affiliate, *Association Camerounaise pour le Marketing Social* (ACMS) has been implementing social franchising, especially for long-term methods. Social marketing activities have also been carried out since the early 1990s, with focus on HIV/STD prevention, family planning, and improved awareness and preventive behaviour.

Data from the 2018 CDHS suggest that mass media promotion of family planning is limited. Almost seven in ten women (69%) and nearly six in ten men (58%) were not exposed in the last 12 months before the survey to messages about family planning. Indeed, they neither saw nor heard messages about family planning through media such as radio, television, newspapers/magazines, cell phone. The percentages of women and men who listened to family planning messages on radio and television are 14% and 21%, respectively, for radio and 18% and 22% for television. Newspapers/magazines and cell phones are uncommon information channels for conveying family planning messages, to both women and men. Overall, then, the population in Cameroon, and postpartum women in particular, have limited access to family planning messaging. It should be noted that an effort is being made through community radio stations to raise awareness of RH/FP, particularly in the most affected regions.

## Methods

This paper uses data from the most recent CDHS conducted in 2018. We define the extended postpartum period as the 18-month period following childbirth, subdivided into three segments: 0–5 months, 6–11 months and 12–17 months. From the merged CDHS women-child datasets, 3101 children aged 0–17 months born to women aged 15–49 years were extracted. For the 28 women who had two births in the 18 months prior to interview, attention is restricted to the more recent birth. Out of the 3073 remaining records, 66 were twins and these were excluded from the analysis. As a result, the dataset for the analysis consisted of 3007 postpartum women, as shown in Table 1. As the 2018 CDHS did not include a contraceptive calendar, we used a current status approach in our analysis. Thus, key variables (contraception, sex, menses) reflect the situation at time of interview. The underlying, and reasonable, assumption of our current status approach is that results obtained from a cohort of women followed from childbirth for 18 months would be very similar to those that we report.

**Table 1:** Characteristics of the sample of postpartum women, 2018 Cameroon DHS

	N	%
Months postpartum		
< 6	1033	34.5
6–11	958	31.5
12–17	1016	34.0
Age		
15–24	1238	38.7
25–34	1346	46.9
35–49	423	14.4
Marital status		
Never married	435	12.1
Currently married	2399	82.5
Formerly married	173	5.4
Number of living children		
< = 1	794	25.7
2	634	20.5
3	488	16.0
4+	1091	37.8
Pregnant		
No	2915	97.1
Yes, intended	72	2.3
Yes, unintended	20	0.6
Want a child in < 12 months		
No	2656	87.8
Yes	351	12.2
Last child is alive		
No	129	3.8
Yes	2878	96.2
Last child wanted		
Wanted then	2271	77.6
Wanted later	606	18.7
Wanted no more	130	3.7
Residence		
Douala/Yaoundé	353	15.4
Other urban	951	27.9
Rural	1703	56.7
Region		
Adamaoua/North/Far-North	1024	40.2
Centre/East/South	1090	29.0
Littoral/West	681	22.9
North-West/South-West	212	7.9
Household wealth		
Poor	1008	37.0
Middle	997	29.6
High	1002	33.4
Education		
None	695	27.4
Primary	939	30.0
Low Secondary	862	25.8
High Secondary+	511	16.8
Religion		
Catholics	1074	35.4
Other Christians	1031	30.6
Muslims/Other	902	34.0
Number of antenatal care (ANC) visits		
0	490	16.5
1–3	688	22.5
4+	1829	61.0
Health facility delivery		
No	928	32.8
Yes	2079	67.2
Number of EPI vaccinations		
0–2	806	26.2
3–7	798	27.6
8–9	1403	46.2
Discussed FP during a health facility visit		
No	2005	67.7
Yes	1002	32.3
Visited by health worker/FP discussed		
No	2096	67.1
Yes, FP not discussed	645	24.6
Yes, FP discussed	266	8.3
N	3007	NA

### Variables

#### Contraceptive use

In this study, modern methods include condom (male or female), other short acting methods (pill, injection, lactational amenorrhea, emergency contraception, standard days method, and other modern methods), and long-acting methods (IUD, implants/Norplant, and sterilization).

#### Postpartum pregnancy risk status

Our definition of exposure to the risk of an unintended pregnancy takes into account the following variables: resumption of sex; resumption of menses; use of a modern or traditional method (mainly withdrawal and periodic abstinence); whether another child was wanted within 12 months; and for the small number of women already pregnant, whether the pregnancy was intended. A woman was defined as not at risk if any of the following conditions applied: sex not resumed, use of a modern method, menses not resumed and child was aged less than 6 months, wanted another child soon or was already pregnant with an intended conception. Among remaining women, two further categories were defined. The fully at-risk category comprised women who had resumed sex and menses, were using no method of contraception or were pregnant with an unintended conception. The residual partially at-risk category included women who had resumed sex but remained amenorrheic with a child aged 6 months or more, and those who were using a traditional method of contraception. Note that this definition assumes that pregnancy is most unlikely for amenorrheic women in the first 6 months postpartum, and that the risk increases but remains low after 6 months [7]. To examine the influence of pregnancy risk on contraceptive use (in Table 3), the variable was redefined by excluding use of a modern method of contraception.

#### Covariates

We use a wide range of demographic and socio-economic variables, as shown in Table 1, some of which require explanation. Cameroon is a very diverse country in terms of ethnicity, religion and environment. The regional variable partially captures this variability. The regions of Adamawa, North and Far-North form the Northern zone where Islam is the main religion, the level of education is relatively low, and child marriage very prevalent. The Centre, East and South regions are home to the Beti ethnic group. Sexual norms are more liberal among this group, with premarital childbearing tolerated and even valued. Residents of the North-West and the West regions share similar values and norms regarding sexuality, marriage and child birth, which are viewed as largely restrictive [21, 22]. However, since the North-West and South-West regions have been experiencing civil unrest for the last five years or so, with consequential disruption of health services, these regions were regrouped together. The Littoral and West regions form the residual group.

Rural–urban residence is represented by three categories. The two major cities, Douala and Yaoundé, each with a population of about 1.3 million, form the first category. The second category comprises other cities and towns, ranging in population size from 5000 to 435,000, and the third comprises the rural population. Given its high correlation with place of residence, the asset-based household wealth variable was removed from the bivariate and multivariate analyses.

The 2018 CDHS asked eight questions related to food security at the household level (e.g., “Did you *worry about not having enough food to eat in the last 12 months?”*). In an attempt to capture another dimension of poverty status at the household level, the answers to these questions were summarized using principal component analysis, and recoded as tertiles. However, the variable did not show any association with the outcomes of interest, and was omitted from the analysis. For women’s education, the secondary level was sub-divided into low secondary (7–10 years of education), and high secondary (11–13 years of education), and the latter combined with the tertiary level.

As expected, antenatal care (ANC) was strongly associated with place of delivery, and both variables were strongly related to child immunization (results not shown). Consequently, only child immunization was retained to capture women’s use of MCH services. Our focus is on the nine Expanded Program on Immunization (EPI) vaccines in Cameroon, namely, Oral Polio vaccine (OPV) 0 (at birth), OPV 1–3, BCG (Tuberculosis), Diphtheria/Tetanus/Pertussis (DPT) 1–3, and Measles. All women were asked whether they had received family planning counselling at any health facility in the past 12 months, and whether they had been visited by a health worker and if so, whether family planning was discussed during the visit. Both these binary variables are included in the analysis.

### Data analysis

Descriptive analyses were first undertaken to examine the distribution and patterns of the variables of interest. Bivariate and multivariate logistic analysis was used to examine the determinants of contraceptive use. The STATA version 15.0 software package was used for the analyses. Sampling weights were used in the descriptive analysis, and the complex sample design used in DHS data collection was accounted for by using the *svy* command in the logistic regression models.

## Results

The characteristics of the sample of postpartum mothers and children are shown in Table 1. The majority of mothers were aged less than 35 years, were currently married with two or more living children and lived in rural areas. A large minority of 40% came from the Northern zone (Adamawa, North and Far North), which, in part, reflects high fertility in this zone. Slightly less than half (43%) had some secondary or higher education. In terms of religion, the sample is divided with approximate equality into Catholics, other Christians and Moslems. With regard to health care, a large majority of 83% had made at least one antenatal visit and two-thirds had delivered in a health facility. Uptake of child vaccinations was high, with over 70% of children having already received three or more vaccinations. One-third of mothers reported discussion of family planning at a health facility visit in the past 12 months, and a similar proportion had received a home visit from a health worker, though family planning was rarely discussed during these visits. Close to 20% of mothers reported that the pregnancy leading to the last birth was mistimed and an additional 4% classified the pregnancy as unwanted. Only 3% were pregnant at the time of interview, though this is likely to be an underestimate because of underreporting of first trimester pregnancies [23].

**Fig. 1:**
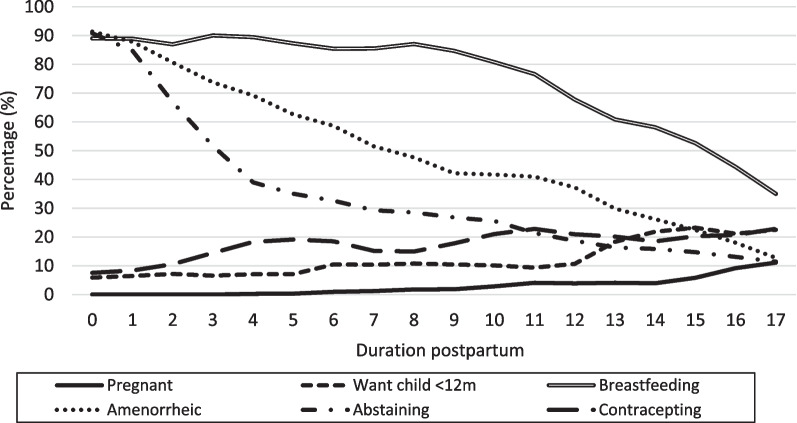
Percentage of women in specific risk of pregnancy states, by duration postpartum

The evolution over the 18 months following childbirth, of behaviour and physiological states that determine risk of conception is shown in Fig. 1. About 90% of mothers breastfed the child in the initial 8 months, after which a steady decline was observed to reach about 35% among mothers at 17 months postpartum. The proportion of women still amenorrheic fell in a linear fashion from 91 to 13%, with a median of 8 months. The proportion still abstaining from sex dropped very steeply from 91% at month 0, to 39% among mothers at a duration of 4 months, with a much gentler decline thereafter. Among mothers at month 17, 11% were still abstaining. Use of a modern contraceptive method rose from 8% shortly after delivery to about 19% among mothers at month 5, but with little further increase among those at longer durations. Desire for another child soon remained very low among mothers with an infant aged 12 months or less, but then rose to about 22% by the end of the observation period. Finally, the percent currently pregnant at month 17 was 11%.

**Fig. 2:**
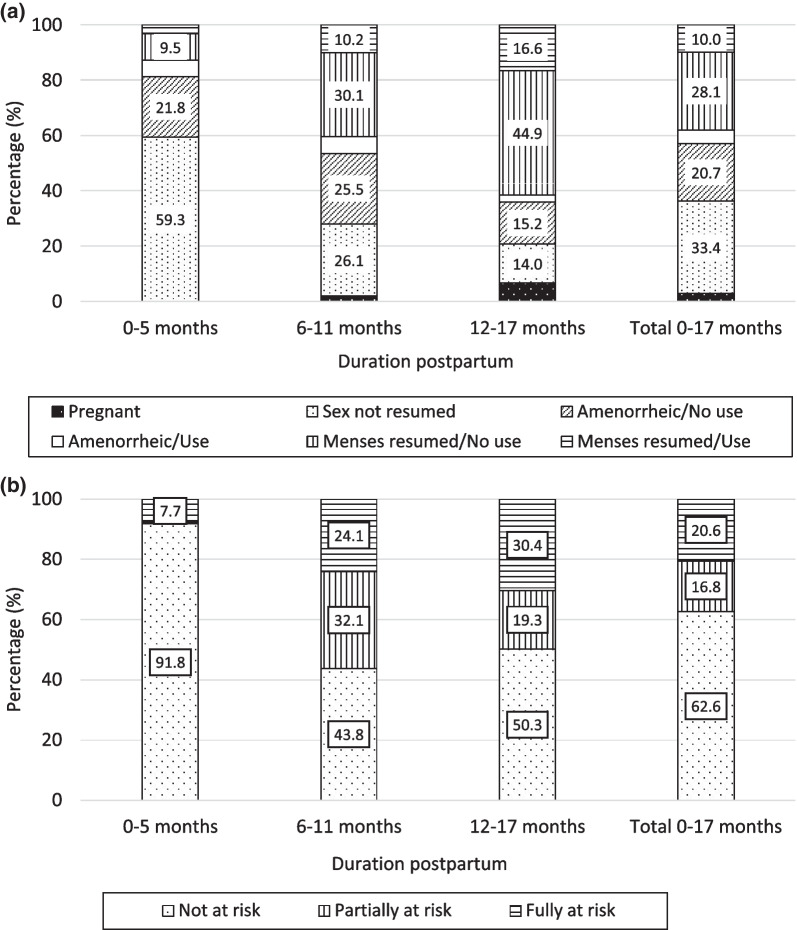
**a** Women’s exposure to pregnancy risks by duration postpartum. **b** Percent of women classified as fully, partially and not at risk of unintended pregnancy

Figure 2a summarises the main determinants of pregnancy risk for three segments of time since birth and provides additional information on overlap of menstrual status and modern method use. The very few women who reported contraceptive use before resumption of sex are subsumed in the “sex not resumed” category, as sexual abstinence is the more effective pregnancy-prevention strategy. In the first six postpartum months, 59% of women had not resumed sex. This fell to 26% at months 6–11 and further to 14% at months 12–17. The corresponding estimates for the category of women who had resumed sex but remained amenorrheic and were not using a modern method were 22%, 25% and 15% over the three segments. Amenorrheic users accounted for a very small fraction of mothers, ranging from 3 to 6%, at all three postpartum durations. The proportions in the category of “sex and menses resumed but no use” rose steeply from 9.5% at months 0–5 months, to 30% in the intermediate segment and to 45% at durations 12–17 months. The proportion who had resumed sex and menses and were using a modern method also rose, but modestly, from 3%, to 10% and finally to 17% at durations 12–17 months.

From these estimates, together with smaller contributions from traditional method use, desire for another child soon, and intended versus unintended current pregnancy, our summary risk status, as defined earlier, was derived (Fig. 2b). At 0–5 months, 92% were classified as not at risk, almost entirely because sex or menses had not started, with a small residue classified as fully at risk. By 6–11 months, the proportion not at risk had fallen to 44% and the proportion fully at risk had risen to 24%. About one-third fell into the partially at risk category; these were largely amenorrheic non-users. At 12–17 months, the proportion fully at risk rose further to 30% but the proportion not at risk also rose to 50%, because of increased contraceptive use, a growing desire for another child soon, and intended current pregnancy.

**Fig. 3:**
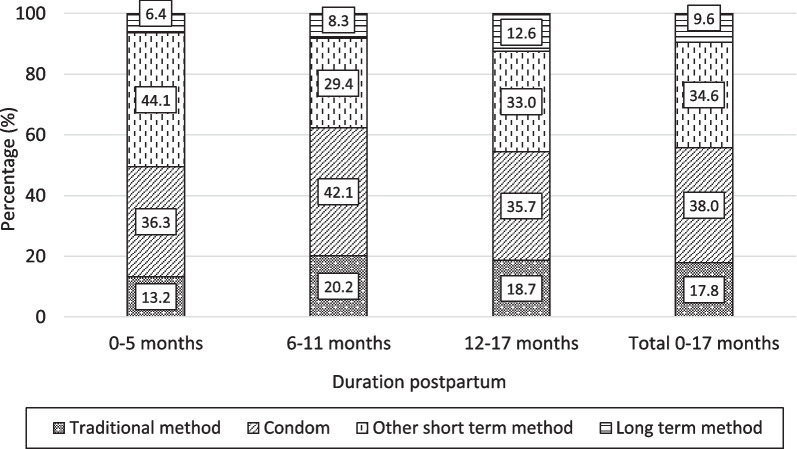
Method-mix by duration postpartum

The method-mix of postpartum users is shown in Fig. 3. Among all postpartum women, condoms accounted for 38.0% of use, followed by other modern short-acting methods, mainly injectables (34.6%). Traditional and long-acting methods comprised 17.8% and 9.6%, respectively, of all use. While the method-mix changed only marginally by postpartum period, the share of long-acting methods rose noticeably from 6% among mothers at months 0–5 to 13% at months 12–17; that of traditional methods also increased slightly from 13 to 19% over the same postpartum durations; and the share of other modern short-acting methods dropped from 44 to 33%.

Table 2 presents a descriptive analysis of risk status and modern method use. Focussing initially on the percent classified as fully at risk of an unintended pregnancy, there were few substantively large and statistically significant differences. Region of residence was one exception. The percentage fully at risk was highest in the North-West and South West zone (26.6%) and lowest in the Centre/East/South (17.5%). Women who had discussed family planning at a health facility visit were less likely than others to be at risk (15.6% versus 23%). The pronounced counterintuitive gradient by number of vaccinations is an artefact of co-variation with age of child.

**Table 2:** Risk of unintended pregnancy and modern contraceptive use, by background variables

	Risk of unintended pregnancy				Modern contraceptive use
	Not at risk	Partially at risk	Fully at risk	Total	
Number of women (unweighted)	1912	478	617	3007	535
Overall	62.6	16.8	20.6	100.0	17.0
Months postpartum	***				***
< = 6	91.8	0.4	7.7	100.0	13.2
6–11	43.8	32.1	24.1	100.0	17.6
12–17	50.3	19.3	30.4	100.0	20.4
Age	Ns				**
15–24	64.4	15.5	20.1	100.0	19.0
25–34	62.0	17.3	20.7	100.0	16.9
35–49	59.6	18.8	21.7	100.0	12.1
Marital status	***				ns
Never married	72.4	7.9	19.7	100.0	19.2
Currently married	60.4	18.6	21.0	100.0	16.7
Formerly married	74.0	10.3	15.8	100.0	16.9
Pregnant	***				***
No	62.1	17.3	20.5	100.0	17.5
Yes	78.1	0.0	21.9	100.0	0.0
Last child is alive	***				ns
No	74.7	5.2	20.1	100.0	12.7
Yes	62.1	17.3	20.6	100.0	17.2
Last child wanted	ns				***
Wanted then	62.1	17.0	20.9	100.0	15.2
Wanted later	64.4	15.1	20.5	100.0	22.1
Wanted no more	63.9	22.1	14.0	100.0	28.6
Residence	***				***
Douala/Yaoundé	67.4	10.1	22.5	100.0	26.7
Other urban	65.6	14.1	20.3	100.0	21.5
Rural	59.8	20.0	20.2	100.0	12.2
Region	***				***
Adamaoua/North/Far-North	56.1	22.0	22.0	100.0	8.0
Centre/East/South	67.7	14.7	17.5	100.0	26.6
Littoral/West	66.9	13.2	19.9	100.0	21.3
North-West/South-West	64.4	9.0	26.6	100.0	15.6
Education	***				***
None	54.9	23.0	22.1	100.0	5.3
Primary	64.9	16.6	18.5	100.0	15.4
Low Secondary	64.4	15.7	19.9	100.0	23.9
High Secondary+	68.1	8.9	23.0	100.0	28.4
Number of EPI vaccinations	***				***
0–2	75.5	9.9	14.6	100.0	8.6
3–7	66.4	15.5	18.2	100.0	14.3
8–9	53.0	21.6	25.4	100.0	23.5
Discussed FP during a health facility visit	***				***
No	60.6	16.4	23.0	100.0	13.8
Yes	66.7	17.8	15.6	100.0	23.7
Visited by health worker/FP discussed	***				***
No	64.1	15.2	20.6	100.0	16.7
Yes, FP not discussed	57.0	20.9	22.1	100.0	14.2
Yes, FP discussed	66.5	17.6	15.9	100.0	28.2

Statistical significance: ***p-value < 0.01; **p-value < 0.05;

EPI: Expanded Program on Immunization

Larger variations were found in the percentages defined as partially at risk, reflecting to a large extent, differences in length of lactational amenorrhea. Only 10% of women in the two major cities were at partial risk compared with 20% in rural areas. Similarly, 9% of those with higher secondary or tertiary education were at partial risk compared with 23% of those with no schooling. Pronounced regional differences were observed, ranging from 9 to 22%. Never- and formerly-married women were less likely to be at partial risk (and more likely to be not at risk) than married women, probably because of differences in resumption of sex.

Large disparities in proportions using a modern contraceptive method were found. Use was highest (27–28%) in Douala/Yaoundé, the Centre/East/South zone and among women with high/secondary/tertiary schooling and lowest (5–12%) in rural areas, in the Adamawa/North/Far North zone and among those with no schooling. The probability of use fell with women’s age. It was higher for those whose most recent birth had been unintended than intended and for women who had discussed family planning in the past 12 months with a health worker compared with those with no such discussion.

Predictors of modern method use were further assessed in Table 3 by logistic regression. We initially ran two multivariate models, first omitting the two variables on family planning discussion and then with their inclusion. As results were closely similar, only those from the second model are shown. With two exceptions (survival of the child and marital status), all factors had statistically significant associations with use at the 95% confidence level, both before and after adjustment. As expected, risk status was strongly predictive of use and adjustment for other factors made little difference. Compared with women classified as not at risk (sex not resumed and/or amenorrheic with an infant aged 0–5 months and/or want another child soon), the adjusted odds ratio of modern method use for those partially at risk (mainly amenorrheic with a child aged 6 or more months) was 2.62 and rose further to 3.24 for those fully at risk.

**Table 3:** Odds ratios from logistic regression of modern contraceptive use among postpartum women

	Unadjusted odds ratios	Adjusted odds ratios
Pregnancy risk status excluding modern contraceptive use		
Not at risk	*Ref*	*Ref*
Partially at risk	2.11***	2.62***
Fully at risk	3.70***	3.26***
Age		
15–24	1.69***	1.63**
25–34	1.48**	1.41
35–49	*Ref*	*Ref*
Marital status		
Never married	*Ref*	*Ref*
Currently married	0.84	1.58***
Formerly married	0.85	1.68*
Last child is alive		
No	*Ref*	*Ref*
Yes	1.42	0.86
Wanted last child		
Wanted then	*Ref*	*Ref*
Wanted later	1.57***	1.18
Wanted no more	2.23***	2.26***
Residence		
Douala/Yaoundé	2.63***	1.25
Other urban	1.98***	1.41**
Rural	*Ref*	*Ref*
Region		
Adamaoua/North/Far-North	*Ref*	*Ref*
Centre/East/South	4.18***	2.26***
Littoral/West	3.12***	1.58**
North-West/South-West	2.13***	1.04
Education		
None	*Ref*	*Ref*
Primary	3.25***	2.03***
Low Secondary	5.59***	2.58***
High Secondary+	7.04***	3.25***
Number of EPI vaccines		
0–2	*Ref*	*Ref*
3–7	1.78***	1.46
8–9	3.28***	1.64**
Discussed FP during a health facility visit		
No	*Ref*	*Ref*
Yes	1.94***	1.39***
Visited by health worker/FP discussed		
No	*Ref*	*Ref*
Yes, FP not discussed	0.83	1.24
Yes, FP discussed	1.96***	1.65**
N	3,007	3,007

Statistical significance: ***p-value < 0.01; **p-value < 0.05

EPI: Expanded Program on Immunization

Among the demographic, social and health-related factors, women’s education was most strongly associated with use. Odds ratios were severely reduced after adjustment but nevertheless rose monotonically to 2.03, 2.59, and 3.27 with ascending level of education compared with the no education category. In contrast, the pronounced urban–rural differences were severely attenuated after adjustment. Very strong associations between region and use approximately halved after adjustment for other factors but, compared to the Adamawa/North/Far North zone, use was higher in Centre/East/South and in Littoral/West zones with adjusted odds ratios of 2.26 and 1.67, respectively. Women who declared their child as unwanted at time of conception had a much higher adjusted odds ratio of use (2.25) than those with an intended pregnancy but, as these women comprised only 4% of the sample this result carries little substantive importance.

Other factors in the model had only modest adjusted associations with modern method use, with odds ratios ranging in magnitude from 1.3 to 1.6. Use was higher in young than older women, among the currently or formerly married than single women, among those whose child had received eight or more vaccinations than those with less than three, and among women who had discussed family planning in the past year (during a health facility visit or a home visit by health worker) than those with no discussion.

## Discussion

In this paper we examined postpartum behavior in a population where fertility remains high, use of modern contraception remains low and where lactational amenorrhea, together with postnatal sexual abstinence, acts as the most important constraint on childbearing within marriage. Our main contribution to the extensive postpartum literature is its focus on the degree to which women in Cameroon are exposed to the risk of an unintended pregnancy in the 18 months following childbirth, considering the resumption of sex and menses as well as contraceptive use. The decision to restrict attention to the 18 months following childbirth was based partly on consideration that protection against pregnancy over this period of time safeguards against the shortest birth intervals that carry the most risk to child survival. At 18 months most women in Cameroon have weaned their child and have resumed menses.

Our definition of risk is contentious, because it departs from the orthodoxy that, following childbirth, sexually active women should use a modern method of contraception if they wish to avoid pregnancy, unless they are observing the conditions of LAM. Specifically, we classified all amenorrheic women with an infant aged less than 6 months as protected against pregnancy risk, regardless of the extent of supplementary feeding. Further, amenorrheic women with a child aged six or more months were classified as partially at risk. The evidence to support these decisions is sparse and dated but nevertheless positive. It is also consistent with the behavior of Cameroonian women, many of whom await the return of menses before starting a method of contraception. At 12–17 months postpartum, for instance, only 14.6% of sexually active amenorrheic women reported use of a modern method compared with 27% of menstruating women. And as shown in the top panel of Table 3, the degree of protection from delayed return of sex and menses was the single biggest predictor of contraceptive use, even after adjustment for other co-variates.

In the definition of risk status, we made an explicit choice to focus on unintended pregnancy rather than all pregnancies. The implication was that women wanting another child soon or within the next 12 months were classified as not at risk. Overall, only 12% wanted another child soon though this proportion was higher among those with a child aged 14 or more months (see Fig. 1). As nearly 90% of our sample wished to delay pregnancy, it is most likely that an analysis based on all pregnancies would have yielded similar results to those reported in this paper. We were also faced with a choice of how to handle the small number of 92 women who reported that they were pregnant. As their exclusion would have introduced a bias, we decided to classify them on the basis of intendedness. Women who declared that the pregnancy was wanted at that time (i.e., intended) were classified, along with those wanting a child soon, as not at risk while others were classified as fully at risk.

Following this classification, we showed how exposure to risk changed over the 18 postpartum months. In the first 6 months after birth, over 90% of women were classified as fully protected, predominantly because of delayed resumption of sex and menses. Reported use of LAM was trivially small at less than 1%. As time since birth lengthened, the protective effect of sexual abstinence and amenorrhea waned and was only partially compensated by a gradual increase in modern method use. The proportion fully at risk (i.e., sex and menses resumed, and no contraceptive use despite a desire to avoid pregnancy) rose from 8% at 0–5 months to 30% among those with a child aged 12–17 months, as shown in Fig. 2b. These estimates are much lower than estimates of unmet need that do not consider the protective effect of abstinence and amenorrhea, except for self-reported use of LAM [4, 10]. At 6–11 months, nearly one-third of women had resumed sex but remained amenorrheic and were not using a modern method and were thus defined as partially at risk.

There are several implications of this evolution for provision of postpartum contraception counselling and services. First, family planning programs should seek to inform women that reliance on lactational amenorrhea carries an unpredictable yet modest risk of conception, but a risk that rises with the duration since birth. Second, while women should be able to choose their preferred method, providers should be aware that provision of short acting hormonal methods, such as pills and injectables, early after childbirth is sub-optimal. High rates of discontinuation of these methods imply that many women will have discontinued use by the time the protective contribution of abstinence and amenorrhea has ceased. As shown in Fig. 3, the method-mix in the first 6 months postpartum is dominated by short-acting methods with a tiny contribution from long-acting ones. Third, counselling about, and provision of, long-acting methods immediately after delivery or in the early postpartum months should be should be part of the strategy. While less than half of married women were aware of IUDs in 2018 and use remains very low in Cameroon, awareness of implants among married women increased from 51 to 72% and use rose from 0.7% to 2.6% between 2011 and 2018. Bearing in mind that two-thirds of births take place in a health facility and that child immunization rates are high, it is probable that this trend could be accelerated by improved availability and counselling. One possible blueprint for action comes from a cluster-randomized trial in Burkina Faso, a country that has similarities with Cameroon in terms of demand for, and use of contraceptive methods. A set of low technology interventions that raised the profile of family planning across the spectrum of MCH services doubled the level of modern method use at 12 months postpartum with an equal share of long- and short-acting methods [24].

The results in Table 2 are instructive and justify our emphasis on a broad definition of risk. As expected, sharp gradients in current use of modern contraception by education and residence were observed. Based on these results, it is tempting to conclude that the need for postpartum contraception is most pressing among the rural, less-educated sectors of the population. However, when sexual abstinence and amenorrhea were considered, a very different picture emerged. The percent classified as fully at risk of an unintended pregnancy varied little by education or residence. Indeed, women with the highest educational attainment and those living in Douala or Yaoundé were most likely to be classified as fully at risk. The reason is clear. The protective effect of amenorrhea lasts much longer among rural, less educated women than among their urban, better educated counterparts. For instance, CDHS data show that the median duration of amenorrhea is 4.3 months in Yaoundé and Douala compared with 10.8 months in rural areas. Thus, more privileged couples in Cameroon are substituting contraception for lactational amenorrhea as their means of birth spacing. But greater resort to modern methods of contraception does not lead to longer median inter-birth intervals lengths, which are close to 30 months in all population strata. Furthermore, women with secondary schooling and those living in the two major cities were more likely than their counterparts to report the most recent birth as mistimed. Specifically, 24% of birth were reported as mistimed in Douala/ Yaoundé compared with 16% in rural areas. Only 7% of women with no education declared the birth as mistimed. This rose to 19%, 27.5% and 22% for women with primary, lower secondary and higher education, respectively (results not shown but available on request). The conclusion is counterintuitive but clear. The need for enhanced postpartum family planning is just as pressing, if not more so, among privileged strata as among the less privileged.

The correlates of modern method use were further explored by logistic regression in Table 3. One objective was to assess the associations between contraceptive practice and use of MCH services and receipt of family planning counselling at a health facility visit. As shown in Table 1, uptake of antenatal, maternity, and immunization services is high in Cameroon. Because of strong associations between use of these three services, only the number of child vaccinations was included in the regressions. After adjustment for risk status, education, residence, and other covariates, a statistically significant but modest association between number of vaccinations and modern method use was found, with odds of 1.64 for women whose child had received eight or more vaccinations, compared with those with less than three. This result is consistent with most studies elsewhere that found a positive association between uptake of MCH services and contraceptive use [11, 25, 26]. Causal interpretation from observational studies is problematic but the evidence from interventions that offered contraceptive advice and supplies at immunization clinics is encouraging, though meagre [27, 28].

Uptake of postpartum contraception may be enhanced by MCH service use indirectly insofar as women gain confidence and trust in modern medical services and staff and directly to the extent that family planning advice is actively offered when women attend facilities for health reasons. Despite frequent use of MCH services by the majority of mothers, only one-third reported discussion of contraception during a health facility visit in the past year. This result implies weak integration of family planning into routine health services and missed opportunities for informing women about contraceptive options. This defect is by no means confined to Cameroon. Similar evidence of missed opportunities has been documented in Senegal, Uganda, Malawi, Nigeria and Ethiopia [29–31]. In Cameroon, discussion was significantly related to contraceptive use but, after adjustment, the association was modest, perhaps a reflection that condoms, the most commonly used method, are widely known and usually obtained from commercial outlets.

We identified a further type of missed opportunity. As shown in Table 1, about one-third (32.9%) of mothers reported a visit from a health worker in the past year, which suggests that outreach services in Cameroon have appreciable coverage. However, at only one-fourth (25.2%) of these visits was family planning discussed, with the consequence that only 8.3% of all women had discussed this topic at a home visit by a health worker. Though contraceptive use was significantly higher among those with such a visit, it is clear that outreach services currently contribute little to family planning uptake.

Most of the other factors included in Table 3 had statistically significant associations with modern method use, after adjustment for risk status and other covariates. Unsurprisingly, the single most powerful correlate was women’s education. It is clear that, even after considering amenorrhea, better educated women have a higher propensity to adopt contraception than the less educated, though, as noted above, this has little effect on birth interval length or on the risk of mistimed pregnancies. The association between urban residence and contraception was less strong than that for education and it is of interest that the large unadjusted difference between rural and urban residents was greatly reduced after adjustment, probably because of the concentration of highly educated couples in the two major cities and other urban areas.

Regional differences are also of note. Unadjusted differences were very large but odds of use attenuated by about 50% after adjustment. Modern method use was lowest in the Northern zone (Adamawa, North, Far North), which is the poorest and least developed part of the country, with an ethnic and religious composition similar to Northern Nigeria where use is also very low. The large unadjusted difference between the Northern zone and the North-West/South-West zone disappeared after adjustment, suggesting that higher contraceptive use in the latter zone reflects other factors such as education and urbanisation. Contraceptive use in the North- and South-West may also have been depressed by civil unrest that has disrupted services. Conversely, the difference between the Northern zone and the Centre/East/South remained large, after adjustment.

There is much international concern that young women face particular problems in accessing contraceptive services and in persuading male partners to adopt contraception, with the consequence that their levels of contraceptive use are lower and unmet need higher than for older women. Such concern is unjustified in Cameroon, where young women were more likely to use a modern method than older women. Adjustment for education and other covariates made little difference to the age gradient in use. The reasons are unclear but two possible explanations can be identified. First, attitudes towards contraception may be more favorable among the younger than older couples. Second, fecundability among older couples will be lower than in the young and hence adequate inter-birth spacing can more easily be achieved without resort to contraception [32].

Slightly over one-tenth of our sample were single (i.e., never married), a reflection of an upward trend in premarital childbearing as age at marriage increased. Before adjustment, there was no difference in contraceptive use by marital status but, after adjustment, single women were significantly less likely to report current use than married women. Interpretation is difficult. Most single mothers are young and the adjustment for age is thus problematic. It is also likely that infrequent sex among women who are unmarried and not cohabiting may contribute to low use.

## Limitations

In the absence of a retrospective monthly calendar, we had to use a current status approach in our analysis. The fundamental assumption is that the collation of behavioral “snapshots” of different women at specified postpartum months will give very similar results to those that would have been obtained from a longitudinal study in which the same women are followed from birth for 18 months. In other words, we have assumed that the postpartum behavior of women who gave birth in, say, June 2018 will be the same as those who gave birth 15 months earlier in February 2017. Provided that the postpartum duration under examination is short (in our study 18 months), we see no reason to question to the validity of this assumption. The danger would have been to analyse a longer postpartum duration, for instance 24 months, because this would have introduced a selection bias against the appreciable proportion of women who have two births in a 24-month period; the results would no longer be representative of postpartum behavior and risk, because of the omission of the earlier short interbirth intervals of those with two births. Our choice of an 18-month observation window minimises the bias: only 28 women out of 3007 had two births in the 18 months preceding the 2018 survey. The incorporation of pregnant women was an additional safeguard against bias.

The current status approach should enhance accuracy of data, because most responses do not rely on memory. The main exception is date of most recent birth. Other potential limitations include underreporting of current pregnancies, the absence of data on abortions and possible underreporting of traditional methods.

## Conclusions and implications

Though our analysis is limited by the lack of calendar data, that prevent examination of the timing of contraceptive adoption and the level of discontinuation, the conclusion is clear. A large majority of women want to avoid pregnancy in the 18 months following childbirth but few take contraceptive precautions. The risk of unintended pregnancy is very low in the first 6 months, because of delayed resumption of sex and amenorrhea, but thereafter about half of women are at partial or full risk. The weaknesses of postpartum family planning services are exposed by the small proportion of women who received contraceptive information and advice. If Cameroon is to achieve its stated goal of raising use of modern contraceptives, enhanced services for postpartum women is probably the optimum strategy because both the subjective need is high among all socio-economic strata and contact with health services is also frequent. What are the most feasible and cost-effective ways of achieving this enhancement? As updating of the 2015–2020 family planning operational plan is currently under consideration, the research evidence in this paper comes at an appropriate time. We outline our recommendations below.

First and foremost, family planning needs to be fully integrated into maternal, neonatal and child health (MNCH) services to reduce missed opportunities across the continuum of care. Our results and international evidence suggest that closer integration will be effective. The continuum of care from a woman’s pregnancy to childbirth and postnatal period provides unique opportunities to reach her with family planning counselling and services [9, 27]. The high level of use of maternal and child health services in Cameroon shows that integration has the potential to reach the majority of mothers and would also be cost-effective and efficient, since it doesn’t require significant increases in staff, supervision or infrastructure, or impose on women the need for additional facility visits [1, 33]. Provider-side challenges to providing high quality contraceptive services to be addressed in the absence of an increase in staff include resistance to new practices, scheduling difficulties, reliance on patients to initiate discussions, and limited communication between primary care providers and specialists [34, 35].

This recommendation is easy to make but less easy to achieve in view of evidence of the heavy work load and weak motivation of health staff [19]. It will require strong political leadership from the Ministry of Health, and perhaps from other government agencies. In this regard, postpartum family planning has a major advantage because of the well-established and substantial benefits for child survival and health of contraception to space births. This health rationale circumvents more controversial discussion of fertility reduction and family size limitation.

Second, strengthen and scale up community service delivery that includes family planning as a key ingredient. In rural and hard-to-reach communities, women face financial and social barriers that delay or inhibit their access to services. Cameroon’s health system remains overwhelmingly facility-based, yet community health workers (CHWs) have been shown to effectively and cost-effectively extend the reach of health services where human resources for health are scarce [36, 37]. Further, as more trusted persons in in some settings, CHWs have the potential to dispel myths and misconceptions surrounding use of contraception. Finally, community-based family planning programs are the most effective (and perhaps the only) strategy to reach women who do not deliver at a health facility. Our results show that only 8% of women received a visit by a health worker during which family planning was discussed, and contraceptive use was significantly higher among these women. In the absence of a vibrant system of CHWs who bring family planning information and services to rural and peri-urban populations, the goal of improving modern contraceptive prevalence may remain elusive.

Third and finally, the range of contraceptive options including LARC methods should be widened. Our results show that long-acting methods accounted for less than 10% of the methods-mix on average, with the share of condoms as high as 38%. Priority should be given to implants, because knowledge and use of this method are already gradually increasing in Cameroon and this method is rapidly becoming a popular option in many African countries [38]. While it would be ideal to also increase access to IUDs, the extra training and logistics required would pose an unrealistic burden at this relatively early stage of contraceptive transition in Cameroon. Even the popularization of implants would entail a major training program, with an equal emphasis on insertion and removal. It is a concern that no information on the number of staffs trained in implant provision is available. What is now needed is a well organised and monitored training program, coupled with demand-creation activities, with the aim of posting at least two skilled staff in all larger hospitals. Convincing evidence exists that nurses and midwives, and even community health workers, can be trained in implant provision [39]. Serious consideration should also be given to reduction of client-fees for implants.

## Data Availability

The demographic and health survey data are publicly available.

## Abbreviations

ACMS: Association Camerounaise pour le Marketing Social
ANC: Antenatal care
CDHS: Cameroon Demographic and Health Survey
CYP: Couple-years of protection
DHS: Demographic and Health Survey
DMPA-SC: Subcutaneous (SC) depot medroxyprogesterone acetate
E2A: Evidence to Action
FP: Family planning
HIV: Human Immunodeficiency Virus
IUD: Intra-Uterine Device
LAM: Lactational Amenorrhea Method
LARC: Long-acting reversible contraceptive
MCH: Maternal and child health
MNCH: Maternal, neonatal and child health
MSH: Management Sciences for Health
PSI: Population Services International
RH: Reproductive Health
STD: Sexually transmitted disease
TFR: Total fertility rate
UNFPA: United Nations Population Fund
USAID: United States Agency for International Development
WHO: World Health Organization
